# From Opinions to Outcomes: Optimizing Survey Methods and Principles Applicable to the Delphi Process in Critical Care Research

**DOI:** 10.1097/CCE.0000000000001438

**Published:** 2026-07-21

**Authors:** Deepa Ramadurai, Vitaly Herasevich, Scott Bolesta, Gretchen M. Brophy, Deyin Doreen Hsing, Philippe R. Bauer, Pooja Amar Nawathe, Robert MacLaren, Ashish K. Khanna, C. Jessica Dine

**Affiliations:** 1 Section of Pulmonary/Critical Care/Sleep Medicine, Department of Medicine, University of Chicago Medical Center, Chicago, IL.; 2 Department of Anesthesiology and Perioperative Medicine, Mayo Clinic, Rochester, MN.; 3 Department of Pharmacy Practice, Nesbitt School of Pharmacy, Wilkes University, Wilkes-Barre, PA.; 4 Department of Pharmacotherapy and Outcomes Science, School of Pharmacy, Virginia Commonwealth University, Richmond, VA.; 5 Department of Pediatrics, Weill Cornell Medicine, New York, NY.; 6 Division of Pulmonary and Critical Care Medicine, Mayo Clinic College of Medicine and Science, Rochester, MN.; 7 Division of Pediatric Critical Care, Cedars Sinai Guerin Children’s, Los Angeles, CA.; 8 Department of Clinical Pharmacy, University of Colorado Skaggs School of Pharmacy and Pharmaceutical Sciences, Aurora, CO.; 9 Department of Anesthesiology, Section on Critical Care Medicine, Wake Forest School of Medicine, Wake Forest Baptist Medical Center, Winston-Salem, NC.; 10 Perioperative Outcomes and Informatics Collaborative, Winston-Salem, NC.; 11 Pulmonary, Allergy, and Critical Care Division, Hospital of the University of Pennsylvania, Philadelphia, PA.; 12 Perelman School of Medicine at the University of Pennsylvania, Philadelphia, PA.

**Keywords:** Delphi methods, expert consensus, questionnaire design, questionnaires and surveys, survey methods

## Abstract

**BACKGROUND.:**

Survey research in critical care has dramatically increased over the past 25 years, and use of surveys within Delphi methods, which provide consensus definitions for heterogeneous disease states, has similarly expanded significantly. It is crucial for survey researchers to understand the nuances of survey development and expectations of information provided by surveys, to use them correctly. Few simple sources exist to guide development considerations to generate high-quality survey research. No studies to our knowledge exist that draw parallels between the principles of survey research as a guide within the Delphi process, as one potential conduit between a steering committee and expert panel.

**METHODS.:**

We present a narrative review of and framework for rigorous design of survey research, highlighting the applicability to the design of surveys administered within Delphi methods.

**RESULTS AND CONCLUSIONS.:**

The methodologic considerations presented for survey research and Delphi methods can inform high-quality critical care research.

**KEY POINTS**

**Question**: What considerations are relevant in the design of survey research?

**Findings:** Pillars of survey research include: 1) assessing appropriateness of the methods to address the prespecified question, 2) attention to detail in question structure, and 3) pilot testing and preparing for analysis. Delphi methods, which are used to create consensus from a group of experts on a given topic, can use surveys as conduits between the steering committee and expert panel, and therefore may consider following these principles of development.

**Meanings**: Survey research may provide rich information from a large sample, whereas Delphi methods provide similarly valuable expert consensus from a small, knowledgeable, and diverse group. Researchers should follow a structured approach to constructing surveys and Delphi methods studies in critical care research.

## BACKGROUND

Survey research involves the quantitative or qualitative assessment of a group of participants, by descriptive, longitudinal, and comparative means. This processes of construction (i.e., encoding) and interpretation (i.e., decoding) are essential to the successful administration and analysis of survey research (1). Strengths of surveys include the relatively short duration of time required for dissemination and response collection, the capacity to include a tailored group of participants, and elicit opinions on a variety of topics within one research tool. In critical care research, survey research can be powerful, given the multidisciplinary team structure and heterogeneous diseases. However, there are important challenges to consider. All questions are intrusive. They require time, thought, and articulation of knowledge from participants. Although every question can be a “learning device,” the few immediate benefits to participants from survey research can present a significant challenge in obtaining adequate responses (1). Furthermore, survey design is a complex, multistep process that requires expertise to ensure that responses accurately reflect the questions being asked.

Survey research is typically used to understand data at a single point in time, sampling a cross-section of a population. Consensus guidelines exist for survey research, with varying recommendations for justification of context and methods, and communication of results (**Table 1**). The broad applicability of survey research and ability to use multiple forms of media contribute to this heterogeneity (2–8). Literature on survey methodology cites existing frameworks available for conduct and analysis of surveys that are clinician-facing and patient-facing (9–11).

**TABLE 1.** A Comparison of Survey Study Development and Reporting Guidelines

Guideline Name	Accurate Consensus Reporting Document	Consensus-Based Checklist for Reporting of Survey Studies	Survey Reporting Guideline	Checklist for Reporting Results of Internet E-Surveys	Preferred Reporting Items for Complex Sample Survey Analysis	Conducting and Reporting Delphi Studies
Application	Reporting guideline for consensus methods	Comprehensive tool for web-based and non-web-based survey research	Guidance on self-administered postal surveys	Reporting data from web-based surveys	Standards for reporting analysis of survey data	Enhance rigor and transparent reporting of Delphi studies
Features	6 sections, 10 subsections, 35 items	6 sections, 19 subsections 30 items	10 groups 33 items	8 sections 30 items	2 sections 17 items	4 sections 16 items
Title or Abstract	Identify the article as reporting a consensus exercise and state	State the word “survey” in title or abstract.	Is the design of the study stated in the title and/or abstract?	NR	NR	NR
	The consensus methods used in the title.	Provide an informative summary.				
Context	Explain why consensus chosen over other approaches.	Rationale, what has been previously done, and why this survey is needed.	Is there an explanation of why the research is necessary, placing the study in context of previous work in relevant fields?	Design Describe the survey design		Rationale Justification for using Delphi technique
	State the aim of the consensus exercise, including audience and geographical scope.	Identify purposes, aims, goals, or objectives.	Is the purpose or aim of the article explained?			
	If exercise is an update, state why the update is needed and provide the original citation.					
Methods	Registration State registration platform and link or that it was not registered.	Study Preparation Describe preparation before conducting the survey (interviewer training, advertising).	Research Tool Is the questionnaire described? If an existing tool was used, are its psychometric properties presented? If an existing tool was used, are references to the original work provided? If a new tool was used, are the procedures used to develop and pretest provided? If a new tool was used, have its reliability and validity been reported? Is a description of the scoring procedures provided?	Development and Pretesting Development and pretesting	Data collection dates Data collection modes Software and code Sample design (stratification, cluster, unequal probabilities)	Planning and Design Explanation of modifications with rigorous and systematic description of application Define consensus a priori
	Selection of SC and/or panelists Describe role(s) and areas of experience or experience of those directing consensus exercise. Explain criteria for panelist inclusion and rationale for numbers. State who was responsible for panelist selection. Describe panelist recruitment. Describe role of any members of public, patients, carers.	Study Design Specify design in the methods section (e.g., cross-sectional, or longitudinal).	Survey Administration Mode of administration? Do the authors provide information on the type of contact and how many attempts were made to contact subjects (i.e., prenotification by letter or telephone, reminder postcard, duplicate questionnaire with reminder)? Do the authors report whether incentives were provided (financial or other)? Is there a description of who approached potential participants (e.g., identification of who signed the covering letter)?	Survey Administration Web/e-mail Context Mandatory/voluntary Incentives Time/Date Randomization of items or questionnaires Adaptive questioning Number of items Number of screens (pages) Completeness check Review step	Target population Sample sizes Public or restricted data	Reporting Explanation of purpose and rationale for Delphi rather than other methods Criteria for selection of experts, nonresponse, and response rates
	Preparatory research Describe summary of existing evidence and provision to panelists. Describe how information was obtained prior to item generation. Describe any systematic literature search in detail. Describe how existing scientific evidence was summarized and provision to panelists.	Data Collection Methods Questionnaire details (e.g., number of sections, number of questions, number and names of instruments). Describe all questionnaire instruments. Report target population reported validity and reliability information, scoring/classification procedure, and references. Provide pretesting information, if performed. Provide the questionnaire.	Sample Selection Is there a description of the survey population and the sample frame used to identify this population? Do the authors provide a description of how representative the sample is of the underlying population? Is a sample size calculation or rationale/justification for the sample size presented?	Recruitment and Sample Description Open vs. closed Contact mode Advertising		Comprehensible methods including preparation, number and design of survey rounds, analysis methods, processing and synthesis of responses
	Assessing consensus Describe methods used and steps taken for panelist input and consensus. Describe how each question or statement was presented and response options. State objectives for each consensus step. State consensus definition. State whether items that met the prespecified definition of consensus were included in any subsequent voting rounds.	Survey Modes of questionnaire administration (type, number of contacts, location of where survey was conducted). Survey time frame (periods of recruitment, exposure, follow-up days). Survey entry process (non-web-based and web-based)				Flowchart to illustrate stages (preparatory, Delphi rounds, interim processing and analysis, and conclusions) Definition for attainment of consensus
	For each step, describe how responses were collected, and whether responses were collected in a group setting or individually. Describe how responses were processed and/or synthesized. Describe any piloting of the study materials and/or survey instruments. Describe how feedback was provided to panelists at the end of each consensus step or meeting State whether anonymity was planned in the study design. State if the steering committee was involved in decisions made by consensus panel.	Sample Characteristics Describe the study population. Describe sampling techniques. Provide sample size, including sample size calculation. Describe sample representativeness of the study population.				
	Participation Describe incentives used to encourage responses or participation. Describe any adaptations to make the surveys/meetings accessible.					
Results	State when the consensus exercise was conducted. Explain any deviations from the study protocol, and why these were necessary.	Report numbers of individuals at each stage of the study. (Consider a flow diagram.) Provide reasons for non-participation at each stage.	Results Is the response rate reported? Are all respondents accounted for?	Response Rates Unique site visitor View rate Participant rate Completion rate	Response rate (including calculation) Missingness rates and methods for handling missing data	Reporting Results reported by rounds (average group response, changes between rounds, modification of survey instrument)
	For each step, report quantitative (number of panelists, response rate) and qualitative (relevant sociodemographics) data to describe the participating panelists	Report and define response rate. Provide definition of unique visitors. Report number of unique visitors and relevant proportions (e.g., view proportion, participation proportion, completion proportion).	Is information given on how nonrespondents differ from respondents? Are the results clearly presented? Do the results address the objective(s)?			
	Report the final outcome of consensus process as qualitative and/or quantitative data. List any items/topics modified or removed.	Provide participant characteristics, confounders, and outcomes. Give unadjusted estimates and, if applicable, confounder-adjusted estimates, 95% CIs, and *p* values.	Discussion Are the results summarized with reference to the study objectives? Are the strengths of the study stated?			
		For multivariable analysis, provide model building process, model fit statistics, and model assumptions. Detail sensitivity analyses performed.	Are the limitations of the study (taking into account potential sources of bias or imprecision) stated?			
			Is there explicit discussion of the generalizability (external validity) of the results?			
Quality Checking		For non-web-based surveys, approaches to minimize human error in data entry.	Ethical Quality Indicators Study funding reported? Research Ethics	Institutional Review Board (IRB) IRB approval Informed consent Data protection	Observation deletion	Study Conduct
		For web-based surveys, provide approaches to prevent “multiple participation” of participants.	Board (REB) review reported? Reporting of subject consent procedures?	Preventing Multiple Entries Cookies used IP check Log file analysis Registration	Suppression rules	Review of information provided to expert panel at the start of the project and throughout the process
		Ethical Considerations Provide information on ethical approval for the study including informed consent, IRB approval, Helsinki declaration, good clinical practice declaration.			Singleton problem (if needed)	Prevent bias, ensure independent researcher coordinates study if there are any conflicts of interest
		Provide information about survey anonymity and confidentiality and describe what mechanisms were used to protect unauthorized access.				Report both consensus and non-consensus to provide insight and highlight differences
		Statistical Analysis Report missing items, missing data mechanism, and methods used to deal with missing data.				External validation by board or authority when complete
		State how nonresponse error was addressed. For longitudinal surveys, state how loss to follow-up was addressed.				Reporting Discussion of limitations
		Other Sections Funding organization roles in design, implementation, and analysis. Potential conflicts of interest.				
		Acknowledgements and contribution to the research.				
Analysis		Describe statistical methods, analytical approach, and the statistical software used for data analysis.	Is the method of data analysis described?	Handing incomplete questions	CIs and SEs	NR
		Report any modification of variables used in the analysis, along with reference (if available).	Do the authors provide methods for analysis of nonresponse error?	Atypical timestamps	Weighting	
		Indicate whether any methods such as weighting of items or propensity scores have been used to adjust for non-representativeness of the sample.	Is the method for calculating response rate provided?	Statistical correction	Variance estimation	
		Describe any sensitivity analysis conducted.	Are definitions provided for complete vs. partial completions?		Subpopulation analysis	
			Are the methods for handling item missing data provided?			
Discussion	Discuss the methodological strengths and limitations of the consensus exercise.	Discuss limitations (potential biases, imprecisions, non-representativeness of sample, study design, important uncontrolled confounders).				Reporting
	Discuss whether the recommendations are consistent with any preexisting literature and, if not, propose reasons why this process may have arrived at alternative conclusions.	Cautious overall interpretation of results, based on potential biases, imprecisions, and suggest areas for future research.				Conclusions with scope and applicability to resulting practice guideline
	List any endorsing organizations involved and their role.	Discuss external validity.				Publication and dissemination with endorsement of guidance by professional associations and healthcare authorities to facilitate implementation
	State any potential conflicts of interests.					
	State any funding received and the role of the funder.					

NR = not reported.

**Figure 1.**
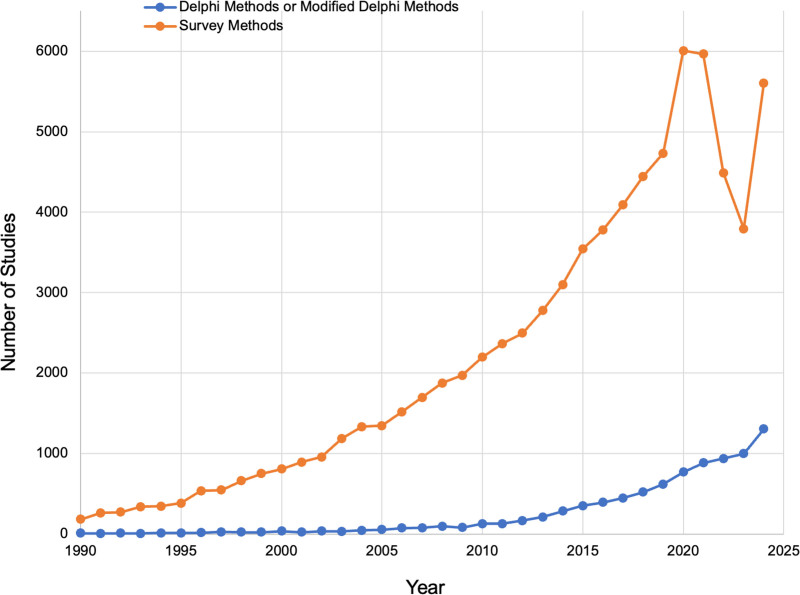
A PubMed search of the number of articles when using the search terms “survey methods AND critical care OR intensive care” (*orange line graph*) and “Delphi methods OR modified Delphi methods AND critical care OR intensive care” (*blue line graph*) from 2000 to 2025.

**Figure 2.**
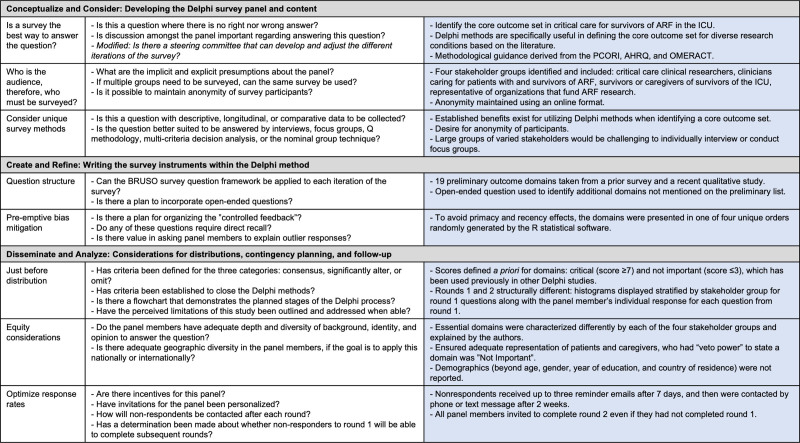
Is the Delphi process right for this question? An assessment using a simplified framework for the approach to survey research and understanding if Delphi methods are appropriate. If any of the “yes-no” questions are answered “no,” or any questions are challenging to answer, reconsider the use of survey methods and Delphi process, or consider clarifying this information before proceeding. The text in italics indicates the question to be asked for “modified” Delphi process. The third column highlighted in *blue* describes an example from Turnbull et al (12), a Delphi methods study published in *Critical Care Medicine* in 2017 to define clinical research priorities for survivors of acute respiratory failure. AHRQ = Agency for Healthcare Research and Quality, ARF = acute respiratory failure, BRUSO = brief, relevant, unambiguous, specific, objective, OMERACT = Outcome Measures in Rheumatology, PCORI = Patient-Centered Outcomes Research Institute.

Importantly, surveys can provide much more than a cross-sectional analysis, especially when integrated or embedded within a broader methodologic framework. Specifically in critical care, the exploration of research needs or clinical applicability of evidence-based practices in heterogenous ICU conditions have increasingly used Delphi methods to understand expert recommendations and applicability of these recommendations to daily ICU care (12–16). Although sharply distinct from traditional cross-sectional survey design, many pearls of survey research design are relevant to the design of Delphi methods research. Despite the critical gap in guidance on developing and ensuring high-quality qualitative research in critical care, use of both cross-sectional surveys and Delphi process research have grown significantly over the past 10 years (**Fig. 1**). We therefore present a narrative review of designing cross-sectional survey research, highlighting fundamental methodologic differences from Delphi methods research (**Fig. 2**), whereas considering how conceptual overlap may exist for surveys embedded within the Delphi design.

## METHODS

### Ethics Approval

This narrative review of methodologic best practices in survey research did not involve human participants or identifiable personal data. In accordance with the University of Chicago Institutional Review Board (Protocol Number 25-2211) and with the Helsinki Declaration of 1975, as most recently amended, this activity did not constitute human subjects research and therefore was exempt from review on January 6, 2026.

### Pillars of Optimal Survey Research

#### Conceptualize and Consider

##### Is a survey the best way to answer the question?

Developing a survey requires grounding in a conceptual framework and a clear definition of its objectives (1). Is the goal of the survey to elicit ideas and opinions? Is a survey the best method to answer the question at hand? If there is little context to the survey research question, it is possible a broader, qualitative exploration may be needed first, to generate hypotheses, and then these hypotheses can subsequently be explored on a survey.

##### Who is the audience, therefore, who must be surveyed?

Survey questions involve implicit and explicit presumptions (1). In asking “What IV fluid should be used in undifferentiated shock?,” we assume 1) the participant believes fluids should be used in undifferentiated shock, 2) the participant understands the differences between undifferentiated shock and other types of shock, and 3) the participant’s fluid choices would differ between these conditions. To best understand the answer, the participants’ baseline education needs to be assessed or standardized. It is imperative to define a hypothesis and potential confounders a priori, as this will affect survey design and distribution.

Physicians, nurses, pharmacists, respiratory therapists, physical and occupational therapists, speech language pathologists, nutritionists, and social workers are all relevant to research questions in the ICU. Furthermore, ICU, hospital, and health system administrators are often stakeholders in critical care operations. Patients and surrogates are historically underrepresented in critical care outcomes research and are primary recipients of critical care services (12). Failure to consider group differences could result in biased data or misrepresentation of certain groups, thus affecting the validity and generalizability of the findings. Sampling can occur by convenience (i.e., a group accessible to the survey administrator) or be purposive (i.e., specific features necessitate inclusion such as selection of ICU directors to better understand system-level ICU workflow). Random, cluster (e.g., by hospital, health system, ICU type, clinical role, etc) or snowball (i.e., select initial participants who then refer others who meet the criteria for participation) sampling may be methodologically appropriate depending on the research question. Sampling must also take into consideration cost, diversity of opinion, bias, and ethics.

##### Ethics considerations

Internationally, there is heterogeneity in ethical mandates for survey research. Some regions do not require formal review protocols for surveys, whereas others do. Challenges therefore arise when an international group is the desired population for a survey study. Given how survey research intentionally assesses opinions and biases with subsequent informational and psychologic harm, full ethics review or exemption procedures are generally recommend before distribution (17, 18). Survey research focused on populations that have been historically marginalized, made vulnerable, or have diminished autonomy, should especially be protected by ethics review (18). The American Association for Public Opinion Research (AAPOR) provides specific guidance with a published code of professional ethics and practices to define terminology used in reference to survey research and standards for disclosure (19).

##### Consider unique survey methods

Surveys may be characterized as: 1) descriptive (i.e., revealing characteristics, behaviors, opinions, and ideas), 2) longitudinal (i.e., tracking numerical or categorical data serially over time), or 3) comparative (i.e., realizing relationships between different variables) (20). Although these basic principles are true, survey research extends beyond a list of questions. Survey research is often thought to be a cross-sectional assessment of a group at a single time point. Surveys, however, can be embedded within a larger methodologic design with a purpose other than to just provide descriptive information. A factorial survey design, for example, is an “experiment within a survey,” using multiple, independent variables randomly incorporated into a clinical vignette, upon which survey questions are based (21). Each independent variable is manipulated to assess how participants respond to questions with different key features. In one example, Quirke et al (22) use the factorial survey design to understand clinician considerations when transitioning from noninvasive to invasive long-term mechanical ventilation via tracheostomy for children with complex medical needs. Using seven independent variables in their vignette, their survey assesses clinicians’ decisions on the initiation of invasive mechanical ventilation. A landmark *New England Journal of Medicine* article used factorial survey design to demonstrate how physicians made disparate decisions on cardiac catheterization for Black women compared with White patients and men, demonstrating bias in clinical decision-making (23). Indeed, there are opportunities with factorial survey design to assess clinical decision-making in one of any heterogenous critical illnesses.

### Conceptualize and Consider: Delphi Methods

Although factorial survey design offers valuable insights into clinical decision-making, the Delphi process may use surveys as a conduit to gather consensus in areas where definitive answers are elusive. Delphi methods use anonymous feedback from participants (i.e., the expert panel), for the development of guidelines or definitions, to resolve controversies in management strategies, create assessment tools, and prioritize needs for future exploration (24, 25). The Delphi process begins with qualitative aggregation of different ideas and solutions for a problem, based on a literature review, focus groups, or preceding related cross-sectional survey responses (25). A foundational assumption of Delphi methods is that participants construct their own ideals and beliefs about a topic through their experiences, as opposed to knowledge diffusion from direct or didactic instruction (6). Each round of the Delphi process is built upon the distribution and completion of a questionnaire or interview. Information sharing from the steering group to the expert panel occurs with surveys, text summaries, medians with interquartile ranges or graphical distributions, and formal reporting of written consensus themes, among other means. Building the survey for a Delphi process requires the same considerations as a cross-sectional survey: questions should be appropriately formatted to mitigate confusion and bias while adhering to ethnical regulations and the audience (expert panel and group who would use the consensus information) should be considered in every aspect of the design (26). Alternative quantitative and qualitative consensus methods should be explored depending on the question to be answered and bandwidth of the research team, including interviews, focus groups, multi-criteria decision, analysis, Q methodology, or the nominal group technique (27).

#### Create and Refine

##### Question structure

Every question in a research survey must follow a clear structure. The “BRUSO” method (i.e., brief, relevant, unambiguous, specific, objective) (1) is easily-applicable: questions must be 20 words or less with three or fewer commas, technical jargon should be eliminated, and vague words that may be interpreted differently by different participants should be eliminated. Something as simple as asking age on a survey can result in unreliable responses, as survey participants generally underestimate their age (28) and drop-down menus of age responses may lead to erroneous reporting (28). Asking date of birth is more accurate, but this is also protected health information and regulatory approval may be affected by changing this seemingly simple question.

Surveys must prioritize limiting cognitive load weighed onto the participant. Seven questions for a Likert scale is preferred, ±2 (29). Demographic questions should be listed at the end of the survey and transitions should be used between sections to ensure logical flow.

##### Response structure

Response presentation and wording are equally as important as the question: they should follow a structure and be mutually exclusive. If a list of responses is presented, the order must be standardized to limit bias of which options are presented before the others. Using alphabetical or chronological lists mitigates implicit persuasion. To limit respondent fatigue, an open-ended response may be considered if prespecified options are not obviously discrete (1).

##### Preemptive bias mitigation

Bias in survey research may be related to or independent of survey design (**Table 2**). Words and phrases selected for question-writing may introduce bias and therefore unintended results (30). Questions should avoid being double-barreled (i.e., questions which ask two or more questions, but only allows one answer), listing double negatives, framing for a suspected answer, leading the participant to the hypothesized answer, being too broad, and using multiple phrases (**Table 3**) (30). Response options should avoid having unbalanced or unmatched response choices compared with what the question is asking. When considering Likert scales, visual analog scales, and adjective scales (**Table 4**), the interpretation of neutral responses (e.g., “neither agree nor disagree”) should be defined a priori or eliminated if potentially confusing (20). Comparative scales with a question stem “What is the difference in…” or “What is the difference between…” may help elicit desired information. Econometric response options use statistical considerations within an observational design to quantify and analyze responses to reveal underlying patterns or causal relationships within observational data. A diverse team of survey methods experts should be engaged early to consider biases that are independent of survey design (i.e., Halo effect, confirmation, selection, and reflexivity) and ensure alignment with objectives.

**TABLE 2.** Common Biases in Survey Research and Ways to Consider Mitigating Bias With Question Structure and Content Before Dissemination

Bias	Definition	How to Address
Dependent on survey design		
Acquiescence	tendency to provide positive response	Use “almost never” or “almost always” rather than “never” or “always” on a Likert scale Definitions may vary across participants (i.e., “frequently”), provide specific numbers and time frames Keep wording neutral—avoid “loaded” wording and stereotypes Consider eliminating neutral responses on Likert scales Consider inserting an attention check: “I am not reading the questions in this survey”
Central tendency	reluctance to respond at extremes	
Deviation	tendency to respond as an outlier	
Nay-saying	tendency to provide negative response	
Recall	more likely to remember what and why vs. where, when, and how	
Social desirability	tendency to present oneself in a favorable light by providing answers one believes will be viewed positively by others	
Ordering effect	responses may differ if questions asked in different order	Perform a pretest
Independent of survey design		
Halo effect	Tendency for positive distortion of attributes	Engage a survey development team (or steering committee for Delphi methods) that has diverse opinions and experiences Actively challenge assumptions made in development of survey questions Look for evidence contradictory to your opinion Acknowledge bias and seek out feedback on survey design and content
Confirmation bias	Tendency to search for and interpret information to confirm preexisting beliefs and/or disregard contradictory evidence	
Selection bias	Systematic error in participant selection resulting in a nonrepresentative population	
Reflexivity bias	Acknowledgement of a researcher’s background and biases shape their study (data collection, analysis, and interpretation)	

**TABLE 3.** Pitfalls Within the Survey Structure

Survey Question Pitfalls	Explanation	Example With Pitfall	Example Corrected
Double-barreled questions	Question asks two questions but allows one response	True or False: Peripherally administered vasoactive agents are efficacious and safe.	True or False: Peripherally administered vasoactive agents are efficacious. True or False: Peripherally administered vasoactive agents are safe.
Double negatives	Using two negatives sequentially to imply the lack of a negative	Should we avoid not using low-tidal volume ventilation in patients with acute respiratory failure?	Should we use low-tidal volume ventilation in patients with acute respiratory failure?
Leading questions	Question that generates a desired answer	How does your hospital incorporate the Surviving Sepsis Guidelines into clinical operations?	Does your hospital use the Surviving Sepsis Guidelines?
Multiple phrases, highly complex, using technical jargon	Too many clauses, too many words	In considering a restrictive or liberal fluid approach to the management of patients with shock and respiratory failure, would you consider the decision to initiate isotonic fluid resuscitation based on pulse pressure variation?	In a patient with shock and respiratory failure, do you use pulse pressure variation to guide fluid resuscitation?
Too broad (ambiguous)	Vague or nonspecific language	Does extracorporeal life support-guided cardiopulmonary resuscitation affect patient outcomes?	Does veno-arterial extracorporeal life support-guided cardiopulmonary resuscitation affect 90-day mortality?

Questions are re-written to demonstrate clarity.

**TABLE 4.**
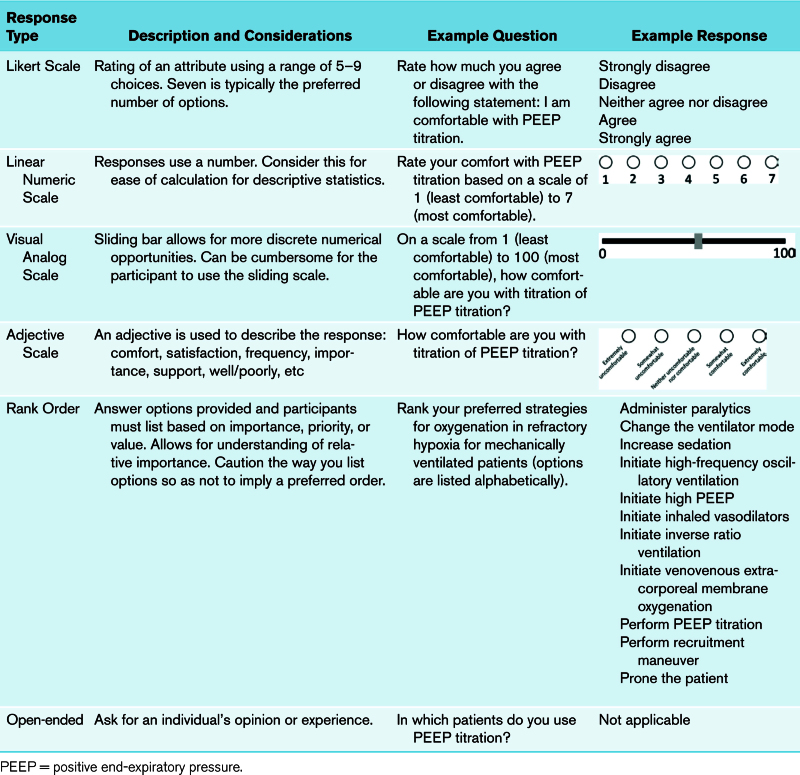
Survey Response Types and Examples

### Create and Refine: Delphi Methods

Surveys are one potential mechanism for information exchange between the steering committee, the group defining and analyzing consensus and disseminating survey rounds, and the expert panel, who votes on each item. Although Delphi methods are not synonymous with conventional cross-sectional survey research, a deep understanding of question development, framing, and structure is essential when building a Delphi methods study.

In each “round” of the Delphi process, the expert panel answers a series of questions. In the first round, experts answer anonymously, using individual knowledge and experiences. On subsequent rounds, experts see “controlled feedback,” which may include descriptive data (e.g., numbers, charts, figures) with measurements of how the entire expert panel responded to each question (24). Open-ended comments from participants may also collected and redistributed to the group. Expert participants therefore trend the group’s responses, compare group responses to their own, and anonymously complete subsequent rounds. This process of iterative and controlled feedback allows for the gradual culling of ideas based on the expert panel opinion toward consensus. The steering committee must determine who participates in each round, what metrics are used to define consensus vs. modification or omission (26). Understanding principles of survey research can be powerful in designing each questionnaire round. The steering committee must make decisions on wording changes and adjusting language not only to meet the recommendations of the expert panel, but to preserve the intention of the consensus report. The BRUSO framework can be applied to the objectives, statements, or questions derived in a Delphi methods study, whereas each statement should also be assessed for potentially biased language. Delphi methods focus on converging expert opinions with controlled feedback, therefore going beyond the point-in-time, cross-sectional estimation of opinions in survey research. However, the way information is created, refined, and communicated to the audience in both forms of research share common goals: to be concise, clear, and unbiased.

#### Disseminate and Analyze

##### Just before distribution

Surveys should be piloted prior to dissemination. A group mirroring the target population and separate from the survey team should complete the survey to provide feedback on content, flow, readability, and length (e.g., subgroup of an ICU operations team, single ICU in a multi-ICU study). Questions specifically eliciting feedback on survey structure should be embedded within the survey pilot.

##### Equity considerations

Surveys should be written at a sixth grade reading level, using a word processing service or online readability resource. Considerations for color blind populations may require changing images and figures. Authors must determine the added benefit vs. logistical challenges of translating surveys into additional languages. The Society of Critical Care Medicine and others have online resources for lexicons that exemplify principles of diversity, equity, and inclusion.

##### Response rate and reliability

Response rate is often the most reported value in survey research to indicate representativeness of the data. Response rates for health sciences surveys range from 20% to 30%, and sometimes up to 40% in education sciences (1, 31). Sending surveys to more participants does not necessarily generate a greater number of responses. A low response rate is often cited as a limiting factor for the publication of survey research. Indeed, when surveys are tailored and explicitly distributed to a select, small, purposive sample, response rates may actually increase (31). Tools to boost survey completion include a visually appealing cover page, monetary incentives, pre-notification by e-mail, directly addressed invitations, reminders, and brevity (i.e., requiring < 10 min to complete) (32).

Methods of assessing the representation of a sample population against the target population include chi-square tests, *t* tests, and analysis of variance. Effect sizes and margin of error can also be used to understand whether the random sample is biased compared with the target population (33).

Surveys should have high internal consistency (i.e., participants should be consistent in their responses), which can be measured by Cronbach alpha in pilot testing. In contrast, inter-rater reliability assesses the way completed surveys are analyzed. Good inter-rater reliability means that the method of analysis consistently produces the same assessment of the results. Cohen kappa for categorical data or Krippendorf alpha for nominal, ordinal, interval, and ratio data are commonly used. Lastly, a correlation coefficient between two scores from surveys completed by the same individual at two different time points reports stability of data over time (34). Additional discussion of survey analysis is beyond the scope of this article, however, should be considered in the early stages of survey design. The AAPOR provides guidance on analyzing and reporting survey results, delineating common methods used, the utility of weighting, transparency, and response rates (19).

### Disseminate and Analyze: Delphi Methods

Two groups must be considered in dissemination of a Delphi methods study: the expert panel and the ultimate audience that will be consumers of the product of the Delphi process. The steering committee may complete any pilot testing of tools to be sent to the expert panel. No consensus exists for expert panel creation. One systematic review identified the minimum, median, and maximum numbers of Delphi process research in the 2011 was 3, 17, and more than 400 (35). The time, energy, and cost of large groups should be taken into account as well as the reproducibility and reliability of the group to deliver consensus on the topic. In general, somewhere between 10 and 15 subjects are suggested if the background of the panel participants is homogenous (26), and up to 20 if there is heterogeneity in the group. Response rates for Delphi studies are classically high, often above 90%, given the small sample and stakeholder role of each expert (25). Experts are ideally cognitively diverse and geographically separated, meeting an internally defined criteria to be an “expert” (e.g., years of clinical experience, authorship in peer-reviewed publications, professional society membership) (20, 25). There is implicit bias in this nonrandom selection and recruitment process. Ensuring that the Delphi panel are regarded as experts is relevant to promote uptake of recommendations by the target audience.

Consensus is typically defined a priori, however, if the objective is to understand a field where little research exists, consensus may be defined as the rounds unfold. Consensus is typically a percent agreement, a central tendency (i.e., median value), or a combination of the two with a range (20). Closing criteria require stability of results from prior rounds with adequate consensus, or after a predefined number of rounds. A minority of Delphi process studies do not define the number of rounds in advance (25), and rely on response stability, reported as a mean and sd for inter- or intra-rater reliability. The concept of stability in Delphi methods research is highly debated, and there is no validated approach for interpreting or defining stability to date (24, 25). In some cases, interviews or focus groups with the expert panel must be conducted to address ongoing debate on statements where consensus is not achieved after multiple rounds. If this occurs, anonymity is lost.

### Details in the Delphi Process

Delphi methods were first used in the Cold War for repeated rounds of feedback and to forecast enemy attacks, and now extend to multiple other disciplines (e.g., economics, finance, and healthcare) for forecasting accurate data (36). Delphi methods maintain anonymity, allowing participants to independently reflect on their responses, removing group conformity (20, 24, 26). With iterative responses, controlled feedback, and rigorously defined a priori statistical response, Delphi methods mean to eliminate any single dominant opinion. Expert panelists must be knowledgeable, with diverse backgrounds and opinions, rather than being representative of a single group. This methodology contrasts with the nominal group technique, where a focus group or cohort is asked to provide opinions aloud with pre-determined semi-structured questionnaires. Although anonymity is lost, open dialogue promotes clarification about language or intention in real time. Semi-structured interviews and focus groups alternatively provide qualitative assessment of language or word choice, phraseology, and nonverbal cues, yet are more time-consuming and limit participant numbers (27).

There are no universally accepted criteria that “modify” a Delphi process. Any deviation from the traditional Delphi process can be considered a modified Delphi: when in-person Delphi methods are conducted and therefore anonymity is lost (20), when a steering committee facilitates discussion (24), or when prespecified items or questions are presented to the expert panel initially, as has been done in critical care research (15, 16, 37–39). When a steering committee facilitates communication, they gather and analyze each round, and create alternative choices to present to the group in subsequent rounds (24). The steering committee thereby pushes the group toward consensus, and balances must be in place to ensure their bias does not drive the results. Steering groups often use many of the same principles for the design of survey research, as communication of the controlled feedback can seriously impact the expert panel contribution in each round. Accurate Consensus Reporting Document (7) and Conducting and Reporting Delphi Studies (6) provide guidance in the choice, design, methods, and reporting of Delphi methods research.

## CONCLUSIONS

The broad scope and clinical heterogeneity of critical care research leaves opportunity for using cross-sectional surveys and Delphi process methods. Cross-sectional survey research elicits opinions from a selected sample of a larger target population, best suited to understand current practice or opinions at a point-in-time. Delphi process research keeps the target population in mind, but probes a smaller, expert panel on opinions, with iterative, controlled, anonymized feedback with the goal of achieving a consensus. Surveys may be the mechanism of information exchange between the steering committee and the expert panel, and therefore conceptualization, creation, and analysis of communication in a Delphi study may have parallel concepts to cross-sectional research. Using a dedicated framework, asking questions through rigorous methodologic frameworks has the promise to significantly enhance objectives and outcomes of opinion-based critical care research.

## ACKNOWLEDGMENTS

Dr. Ramadurai acknowledges the Discovery Research Education Workgroup for supporting the inception and development of this article, including a review of early versions.

## References

[1] Peterson RA. Constructing effective questionnaires. SAGE Publications, Inc.; 2000:3–9

[2] Sharma A Minh Duc NT Luu Lam Thang T: A consensus-based checklist for reporting of survey studies (CROSS). J Gen Intern Med 2021; 36:3179–3187 33886027 10.1007/s11606-021-06737-1 PMC8481359

[3] Grimshaw J. SURGE (The SUrvey Reporting GuidelinE). *In*: Moher D Altman DG Schulz KF Simera I Wager E, eds. Guidelines for reporting health research: A user’s manual. 1st ed. Wiley; 2014:206–213

[4] Eysenbach G: Improving the quality of Web surveys: The Checklist for Reporting Results of Internet E-Surveys (CHERRIES). J Med Internet Res 2004; 6:e34 15471760 10.2196/jmir.6.3.e34 PMC1550605

[5] Seidenberg AB Moser RP West BT: Preferred reporting items for complex sample survey analysis (PRICSSA). J Surv Stat Methodol 2023; 11:743–757

[6] Jünger S Payne SA Brine J: Guidance on Conducting and REporting DElphi Studies (CREDES) in palliative care: Recommendations based on a methodological systematic review. Palliat Med 2017; 31:684–706 28190381 10.1177/0269216317690685

[7] Gattrell WT Logullo P Zuuren EJ van: ACCORD (ACcurate COnsensus Reporting Document): A reporting guideline for consensus methods in biomedicine developed via a modified Delphi. PLoS Med 2024; 21:e1004326 38261576 10.1371/journal.pmed.1004326 PMC10805282

[8] Bennett C Khangura S Brehaut JC: Reporting guidelines for survey research: An analysis of published guidance and reporting practices. PLoS Med 2010; 8:e1001069 21829330 10.1371/journal.pmed.1001069 PMC3149080

[9] Burns KEA Duffett M Kho ME; ACCADEMY Group: A guide for the design and conduct of self-administered surveys of clinicians. CMAJ 2008; 179:245–252 18663204 10.1503/cmaj.080372 PMC2474876

[10] McColl E Jacoby A Thomas L: Design and use of questionnaires: A review of best practice applicable to surveys of health service staff and patients. Health Technol Assess 2002; 5:21–60 10.3310/hta5310 11809125

[11] Rattray J Jones MC: Essential elements of questionnaire design and development. J Clin Nurs 2007; 16:234–243 10.1111/j.1365-2702.2006.01573.x 17239058

[12] Turnbull AE Sepulveda KA Dinglas VD: Core domains for clinical research in acute respiratory failure survivors: An International Modified Delphi Consensus Study. Crit Care Med 2017; 45:1001–1010 28375853 10.1097/CCM.0000000000002435 PMC5433919

[13] Kansal A Latour JM See KC: Interventions to promote cost-effectiveness in adult intensive care units: Consensus statement and considerations for best practice from a multidisciplinary and multinational eDelphi study. Crit Care 2023; 27:487 38082302 10.1186/s13054-023-04766-2 PMC10712165

[14] Pearl JS Gajic O Dong Y: Creation of the prevention of organ failure checklist. A multidisciplinary approach using the Modified Delphi Technique. Ann Am Thorac Soc 2016; 13:910–916 26933899 10.1513/AnnalsATS.201509-626BC PMC5018924

[15] Allum L Pattison N Connolly B: Codesign of a quality improvement tool for adults with prolonged critical illness: A Modified Delphi Consensus Study. Crit Care Explor 2024; 6:e1146 39263382 10.1097/CCE.0000000000001146 PMC11390055

[16] Jaworska N Makuk K Krewulak KD: A National Modified Delphi Consensus Process to prioritize experiences and interventions for antipsychotic medication deprescribing among adult patients with critical illness. Crit Care Explor 2022; 4:e0806 36506828 10.1097/CCE.0000000000000806 PMC9722588

[17] Zimba O Gasparyan AY: Designing, conducting, and reporting survey studies: A primer for researchers. J Korean Med Sci 2023; 38:e403 38084027 10.3346/jkms.2023.38.e403 PMC10713437

[18] Whicher D Wu AW: Ethics review of survey research: A mandatory requirement for publication? Patient 2015; 8:477–482 26392006 10.1007/s40271-015-0141-0

[19] Best practices for survey research. AAPOR; November 10, 2022. Available at: https://aapor.org/standards-and-ethics/best-practices/. Accessed March 1, 2026

[20] Shang Z: Use of Delphi in health sciences research: A narrative review. Medicine (Baltim) 2023; 102:e32829 10.1097/MD.0000000000032829 PMC9936053 36800594

[21] Aguinis H Bradley KJ: Best practice recommendations for designing and implementing experimental vignette methodology studies. Organ Res Methods 2014; 17:351–371

[22] Quirke MB Alexander D Masterson K: Development of a factorial survey for use in an international study examining clinicians’ likelihood to support the decision to initiate invasive long-term ventilation for a child (the TechChild study). BMC Med Res Methodol 2022; 22:198 35864457 10.1186/s12874-022-01653-2 PMC9306171

[23] Schulman KA Berlin JA Harless W: The effect of race and sex on physicians’ recommendations for cardiac catheterization. N Engl J Med 1999; 340:618–626 10029647 10.1056/NEJM199902253400806

[24] Nasa P Jain R Juneja D: Delphi methodology in healthcare research: How to decide its appropriateness. World J Methodol 2021; 11:116–129 34322364 10.5662/wjm.v11.i4.116 PMC8299905

[25] Niederberger M Spranger J: Delphi technique in health sciences: A map. Front Public Health 2020; 8:457 33072683 10.3389/fpubh.2020.00457 PMC7536299

[26] Taylor E: We agree, don’t we? The Delphi Method for health environments research. HERD 2020; 13:11–23 10.1177/1937586719887709 31887097

[27] Mukherjee N Zabala A Huge J: Comparison of techniques for eliciting views and judgements in decision‐making. Methods Ecol Evol 2018; 9:54–63

[28] (PDF) Asking the age question in mail and online surveys. Available at: https://www.researchgate.net/publication/238103842_Asking_the_Age_Question_in_Mail_and_Online_Surveys. Accessed July 25, 2024

[29] Miller GA: The magical number seven plus or minus two: some limits on our capacity for processing information. Psychol Rev 1956; 63:81–97 13310704

[30] Choi BCK Pak AWP: A catalog of biases in questionnaires. Prev Chronic Dis 2005; 2:A13 PMC1323316 15670466

[31] Wu MJ Zhao K Fils-Aime F: Response rates of online surveys in published research: A meta-analysis. Comput Hum Behav Rep 2022; 7:100206

[32] Sammut R Griscti O Norman IJ: Strategies to improve response rates to web surveys: A literature review. Int J Nurs Stud 2021; 123:104058 34454334 10.1016/j.ijnurstu.2021.104058

[33] Ochsner M. Representativeness of surveys and its analysis. Published online December 2021. Available at: https://forscenter.ch/wp-content/uploads/2021/12/forsguides_representativity_v18_final_sub_v03_combined.pdf. Accessed March 24, 2025

[34] Chiang ICA Jhangiani RS Price PC. Reliability and validity of measurement. Published online October 13, 2015. Available at: https://opentextbc.ca/researchmethods/chapter/reliability-and-validity-of-measurement/. Accessed March 24, 2025

[35] Boulkedid R Abdoul H Loustau M: Using and reporting the Delphi method for selecting healthcare quality indicators: A systematic review. PLoS One 2011; 6:e20476 21694759 10.1371/journal.pone.0020476 PMC3111406

[36] Dalkey N Helmer O: An experimental application of the Delphi method to the use of experts. Manage Sci 1963; 9:458–467

[37] Sudore RL Lum HD You JJ: Defining advance care planning for adults: A consensus definition from a Multidisciplinary Delphi Panel. J Pain Symptom Manage 2017; 53:821–832.e1 28062339 10.1016/j.jpainsymman.2016.12.331 PMC5728651

[38] Fiest KM Krewulak KD Makuk K: A Modified Delphi Process to prioritize experiences and guidance related to ICU restricted visitation policies during the coronavirus disease 2019 pandemic. Crit Care Explor 2021; 3:e0562 34712955 10.1097/CCE.0000000000000562 PMC8547909

[39] Haaksma ME Smit JM Boussuges A: EXpert consensus On Diaphragm UltraSonography in the critically ill (EXODUS): A Delphi consensus statement on the measurement of diaphragm ultrasound-derived parameters in a critical care setting. Crit Care 2022; 26:99 35395861 10.1186/s13054-022-03975-5 PMC8991486

